# A Combined Bioinformatics and Clinical Validation Study Identifies *MDM2*, *FKBP5* and *CTNNA1* as Diagnostic Gene Signatures for COPD in Peripheral Blood Mononuclear Cells

**DOI:** 10.3390/ijms27010273

**Published:** 2025-12-26

**Authors:** Innokenty A. Savin, Aleksandra V. Sen’kova, Andrey V. Markov, Olga S. Kotova, Ilya S. Shpagin, Lyubov A. Shpagina, Valentin V. Vlassov, Marina A. Zenkova

**Affiliations:** 1 Institute of Chemical Biology and Fundamental Medicine, Siberian Branch of the Russian Academy of Sciences, Lavrent’ev Avenue 8, 630090 Novosibirsk, Russia; savin_ia@1bio.ru (I.A.S.); andmrkv@gmail.com (A.V.M.); vvv@1bio.ru (V.V.V.); marzen@1bio.ru (M.A.Z.); 2 Department of Hospital Therapy and Medical Rehabilitation, Novosibirsk State Medical University, Krasny Prospect 52, 630091 Novosibirsk, Russia; ok526@yandex.ru (O.S.K.); mkb-2@yandex.ru (I.S.S.); lashpagina@gbuzgkb2.ru (L.A.S.)

**Keywords:** chronic obstructive pulmonary disease, bioinformatics, differentially expressed genes, biomarkers

## Abstract

Chronic obstructive pulmonary disease (COPD) is often diagnosed after significant lung damage has already occurred, highlighting a need for minimally invasive biomarkers for early detection of COPD development. This study aims to identify transcriptional biomarkers in peripheral blood mononuclear cells (PBMCs). A Weighted Gene Co-Expression Network Analysis (WGCNA) was performed on the GSE146560 transcriptomic dataset. Hub genes were cross-validated using independent transcriptomic data (GSE94916), topology analysis of a COPD-related protein–protein interaction (PPI) network, and a text-mining approach. The top candidate genes were validated using RT-qPCR in a clinical cohort, consisting of 28 COPD patients and 13 healthy volunteers, and their diagnostic value was evaluated using receiver operating characteristic (ROC) analysis. WGCNA identified four gene modules significantly correlated with COPD, the functional annotation of which revealed their enrichment in immune and tissue remodeling pathways. Further analysis of the PPI network topology structure and gene expression revealed a hub gene signature that was significantly upregulated in PBMCs of COPD patients, including *MDM2* (6.3-fold, *p* < 0.001), *FKBP5* (7.0-fold, *p* < 0.001), and *CTNNA1* (10.0-fold, *p* < 0.001). ROC analysis demonstrated high diagnostic accuracy for these genes, with AUC values of 0.849, *p* < 0.001, for *MDM2*, 0.957, *p* < 0.001, for *FKBP5*, and 0.958, *p* < 0.001, for *CTNNA1*. *MDM2*, *FKBP5*, and *CTNNA1* represent promising, readily accessible PBMC biomarkers for COPD diagnosis.

## 1. Introduction

Chronic obstructive pulmonary disease (COPD) remains one of the most prominent causes of morbidity and the third leading cause of mortality worldwide [1]. COPD is characterized by persistent respiratory symptoms and progressive airflow limitation due to airway and/or alveolar abnormalities caused by chronic inflammation and the accumulation of inflammatory cells in multiple lung areas [2,3]. This persistent inflammation gradually leads to the obstruction of small airways and to the destruction of lung parenchyma, and, as a result, lung function declines [4]. It should be noted that COPD is a heterogeneous lung disorder with complex etiology caused by significant exposure to noxious particles or gases, primarily tobacco smoke, as well as air pollution, genetic susceptibility, and social environment [5,6].

Diagnosis of COPD relies on medical history, symptom assessment, and pulmonary function tests (PFTs), primarily spirometry [7]. However, in the initial stages of the disease, particularly when COPD develops earlier in life with muted symptoms, functional limitations, and/or structural abnormalities, its identification in the clinic remains challenging and requires new diagnostic and/or prognostic approaches to increase the speed and accuracy of the diagnosis [8]. Biomarkers reflecting unique biological mechanisms of COPD development, including neutrophilic inflammation, eosinophilic airway involvement, or α_1_ antitrypsin deficiency, are progressively being implemented for patient stratification and guidance of targeted therapies, including inhaled corticosteroids or biologics [9]. Currently, four distinct groups of biomarkers are used in the diagnosis of COPD: circulating, sputum-derived, lung tissue-derived markers, and COPD-associated features revealed by high-resolution computed tomography (HRCT). Considering this, circulating and sputum biomarkers are of particular interest as non-invasive diagnostic and prognostic approaches [10]. Among circulating biomarkers, several have proven useful in reflecting systemic inflammation in COPD [11], including peripheral eosinophil count, C-reactive protein, elevated plasma fibrinogen, soluble receptor for advanced glycation end products (sRAGE), surfactant protein D (SP-D) and Clara cell protein 16 (CC16), as well as IL-6 and IL-8. Sputum biomarkers relevant to COPD diagnosis can be divided into two main groups: those specific to neutrophilic COPD (the eosinophil-to-neutrophil ratio, elevated levels of IL-8, IL-1β, and TNFα) and those specific to its eosinophilic variant (eosinophil cationic protein (ECP) and periostin, an IL-13-induced protein) [12,13,14].

Lung tissue-derived biomarkers have attracted considerable attention due to their unique capacity to reflect direct pathological characteristics at disease sites [15]. For instance, elevated activity of matrix metalloproteinases (MMPs) in the lung parenchyma, accumulation of elastic fibers and products of their degradation, or infiltration ratios of specific immune cells (e.g., CD8 T lymphocytes and M1 macrophages) demonstrate direct correlations with pathological progression of small airway fibrosis [16]. However, clinical implementation remains constrained by risks associated with invasive biopsies. Imaging biomarkers of COPD are primarily represented by structural alterations in the pulmonary parenchyma, airways, and vascular system [17]. HRCT is the primary modality for evaluating COPD, with characteristic findings encompassing emphysema, airway remodeling, and pulmonary vascular abnormalities. However, these features represent pathological changes that have already occurred in diseased lungs, making HRCT challenging for diagnosing COPD in its early stages [18].

Among diagnostic candidates sensitive to the early manifestations of COPD, genetic and epigenetic markers are now being actively investigated [19]. These include genetic alterations, such as specific gene polymorphisms, dysregulation of the protease–antiprotease balance, and variants in FAM13A affecting WNT/β-catenin signaling and hedgehog signaling [20], as well as epigenetic signatures, including DNA methylation patterns, histone modifications, and microRNA dysregulation [21,22]. They are considered among the most promising biomarkers for susceptibility and phenotypic characterization of COPD since they reflect the development of pathogenetic mechanisms contrary to the aforementioned biomarkers, which are the result of an already developed disease, though, currently, they are utilized only as investigative tools. A primary barrier to their clinical adoption is the requirement for lung tissue specimens obtained primarily during bronchoscopy or surgery, which are associated with invasive biopsy risks as well as severe concomitant lung disorders (e.g., lung adenocarcinoma or lung squamous cell carcinoma) [23,24].

In this context, peripheral blood mononuclear cells (PBMCs) are a highly promising alternative. PBMCs, including lymphocytes (T, B, and NK cells) and monocytes, play a central role in immune system function and are directly involved in COPD-associated inflammation and fibrosis of the airways and lung tissue. Therefore, they can be considered as a promising source for novel COPD biomarkers, including genetic characteristics [25]. Since PBMCs can be more easily and safely isolated from peripheral blood than biomaterials obtained from lung biopsies, they are considered a promising platform for non-invasive COPD diagnosis. Supporting this potential, Riou et al. demonstrated that analyzing the mitochondrial oxygen consumption rate in PBMCs can effectively identify COPD patients [26]. Furthermore, since COPD is not always accompanied by sufficient sputum secretion [27], the potential of PBMCs for noninvasive COPD diagnosis is evident.

Therefore, the aim of this study was to identify and validate novel COPD biomarker candidates using PBMC gene expression profiling. We applied a comprehensive bioinformatics pipeline, including Weighted Gene Correlation Network Analysis (WGCNA) and topology analysis of a PPI network, to independent publicly available transcriptomic datasets of PBMCs from COPD patients. The most promising gene candidates revealed by in silico analysis were subsequently validated using PBMCs obtained from a clinical cohort. This strategy aims to establish conveniently sourced transcriptional biomarkers for COPD development.

## 2. Results

### 2.1. Identification of COPD-Associated Gene Co-Expression Modules

**Figure 1:**
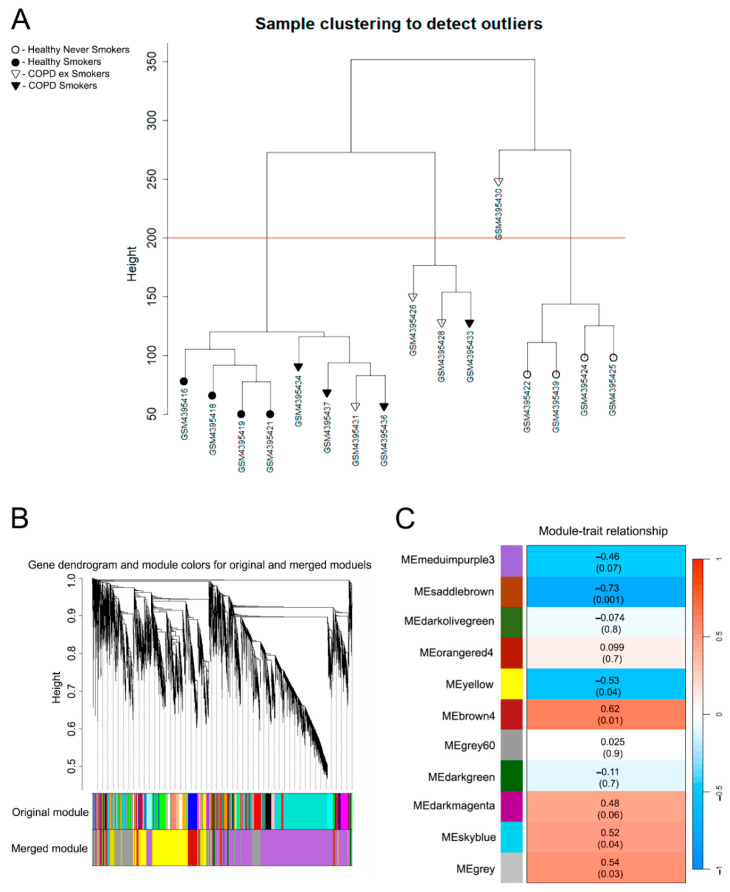
Results of WGCNA of the transcriptomic data obtained from COPD patients and healthy controls. (**A**) Hierarchical clustering (Euclidean distances) of the samples obtained from GSE146560 using gene expression. The red line indicates the cutoff height to remove the outlier sample GSM4395430 due to the aberrant. (**B**) Gene dendrogram demonstrating identified co-expression modules. Each color represents one co-expression module. Original modules with more than 75% similarity were merged, yielding 11 modules. (**C**) Heatmap of the correlation between module eigengenes and COPD status. The color of each cell indicates the correlation coefficient (Pearson). Four modules selected for subsequent analysis are marked in red.

To identify gene networks associated with COPD, we performed a Weighted Gene Co-Expression Network Analysis (WGCNA) on the GSE146560 transcriptomic dataset derived from PBMCs of COPD patients. Initial hierarchical clustering of samples based on Euclidean distance identified one outlier sample (GSM4395430), which was removed from subsequent analysis (Figure 1A). A soft-thresholding power of 13 was selected to achieve a scale-free topology fit (R^2^ > 0.8) while maintaining a high mean connectivity (Figure S1). This yielded a set of co-expression modules that were subsequently merged based on a correlation threshold of >0.75, resulting in a final set of 11 distinct modules, each represented by a unique color (Figure 1B).

We next correlated the module eigengenes (MEs), which represent the dominant expression patterns within each module, with the clinical trait of COPD. To identify genes with diagnostic potential independent of smoking status, we defined a single binary clinical trait for this analysis—presence (1) or absence (0) of COPD diagnosis. Four modules, namely, brown4, darkmagenta, skyblue, and grey, demonstrated moderate to strong positive correlations with the disease status (r = 0.62, 0.48, 0.52, and 0.54, respectively), identifying them as modules of interest for further investigation (Figure 1C). Although the darkmagenta module demonstrated a moderate positive correlation (r = 0.48) with COPD diagnosis, its significance did not meet the conventional significance threshold (*p* = 0.06). However, due to the fact that the darkmagenta module contained a significant part of the analyzed genes, and that the candidate genes would be independently validated both in silico and in a clinical cohort, we decided to include them in the downstream analysis, despite the *p*-value being over the threshold (Figure 1C). Additionally, modules exhibiting a statistically significant negative correlation with COPD were excluded, since detection of up-regulation is considered a more reliable and accessible metric for identifying potential biomarkers than down-regulation.

**Figure 2:**
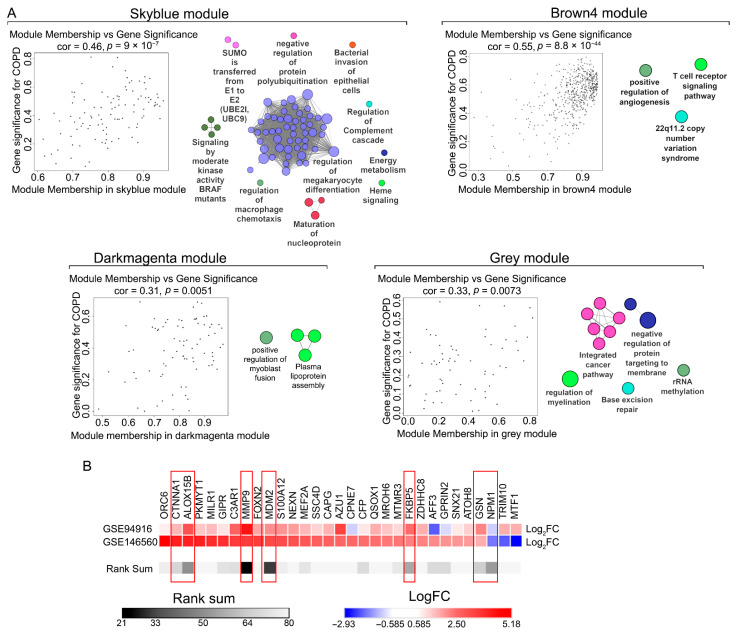
WGCNA modules analysis and selection of genes for validation. (**A**) (**Left panels**): Scatter plots demonstrating correlation between gene intramodular connectiveness (module membership) and expression in COPD (gene significance) for four selected modules. The correlation coefficient was calculated by the Pearson method. (**Right panels**): Functional analysis of genes from each of the selected modules, performed using the ClueGO plugin in Cytoscape software v.3.10.3. (**B**) Heatmap demonstrating expression levels of genes from selected WGCNA modules in two transcriptomic datasets (GSE146560, used in the WGCNA analysis, and independent GSE94916) and their centrality in the STRING network constructed from GWAS-annotated DEGs from the GSE146560 dataset and analyzed by the CytoHubba plugin. Rank sum was calculated as the sum of the degree and bottleneck ranks. Genes selected for validation are marked in red.

To assess the internal coherence and clinical relevance of these modules, we analyzed the relationship between module membership (MM), defined as the correlation between the expression of each gene in the analyzed module and the expression profile of the corresponding eigengene, and gene significance (GS), defined as the correlation between the expression of genes and COPD. Genes within the skyblue and brown4 modules showed moderate positive correlations between MM and GS, indicating that highly connected genes within these modules were strongly associated with COPD (r = 0.46 and 0.55, respectively) (Figure 2A, left panels). These findings confirm that these four modules are enriched for genes biologically relevant to COPD.

### 2.2. Functional Enrichment Analysis of Key Modules

To elucidate the biological processes associated with the COPD-correlated modules, we performed functional enrichment analysis using the ClueGO plugin. Genes from each module were analyzed for enrichment in the Gene Ontology (GO) biological processes, KEGG, Reactome, and WikiPathways databases.

The skyblue module was associated with the greatest number of significantly enriched terms. While the enriched pathways were not exclusively specific to COPD, they were closely related to key disease mechanisms. These included immune system modulation, such as the regulation of macrophage chemotaxis and the complement cascade, as well as processes involved in extracellular matrix remodeling. Similarly, the brown4 module was enriched for terms like the T cell receptor signaling pathway, and the darkmagenta and grey modules showed enrichment in processes related to angiogenesis, myoblast fusion, and protein targeting (Figure 2A, right panels). Collectively, these results suggest that the genes within these modules participate in immune dysregulation and tissue remodeling pathways that are central to COPD pathophysiology.

### 2.3. Selection of Candidate Genes for Clinical Validation

To prioritize candidate gene selection for experimental validation on clinical material, we applied a two-tiered selection strategy. First, we cross-referenced the genes from our four key WGCNA modules with an independent dataset (GSE94916) (Table S1) to identify genes that were consistently and uniformly differentially expressed in PBMCs from COPD patients versus healthy controls (Figure 2B, upper row). Second, we constructed a protein-protein interaction (PPI) network from the differentially expressed genes identified in GSE146560 (Figure S2, Table S2) and analyzed it using the CytoHubba plugin in Cytoscape to reveal hub genes based on high degree and bottleneck scores (Figure 2B, lower row). The rank sum parameter indicates the comparative ranking of a gene within the interactome of the analyzed dataset (lower rank indicates higher interconnectivity).

This integrated approach identified seven candidate genes: *CTNNA1*, *ALOX15B*, *MMP9*, *MDM2*, *FKBP5*, *GSN*, and *NPM1*. Since matrix metalloproteinase 9, encoded by *MMP9* is known to be primarily active in tissues and its functional relevance in PBMCs is unclear, *MMP9* was excluded from further validation. The remaining six genes, namely, *CTNNA1*, *ALOX15B*, *MDM2*, *FKBP5*, *GSN*, and *NPM1*, were selected for downstream validation in a clinical cohort.

### 2.4. Clinical Cohort Characterization

To validate the findings of the bioinformatics analysis, we recruited 28 COPD patients, with an average age of 68.5 ± 10.7 years and a gender distribution as follows: male—64.2% and female—35.7%. COPD severity was assessed using GOLD (Global Strategy for the Diagnosis, Management and Prevention of COPD) 2025 guidelines (https://goldcopd.org/). The distribution of patients by disease severity was as follows: GOLD stage I: 25%, stage II: 21.4%, stage III: 32.1%, and stage IV: 21.4%. For the control group, 13 age- and gender-matched healthy volunteers were recruited.

### 2.5. Validation of Candidate Gene Expression in a Clinical Cohort

**Figure 3:**
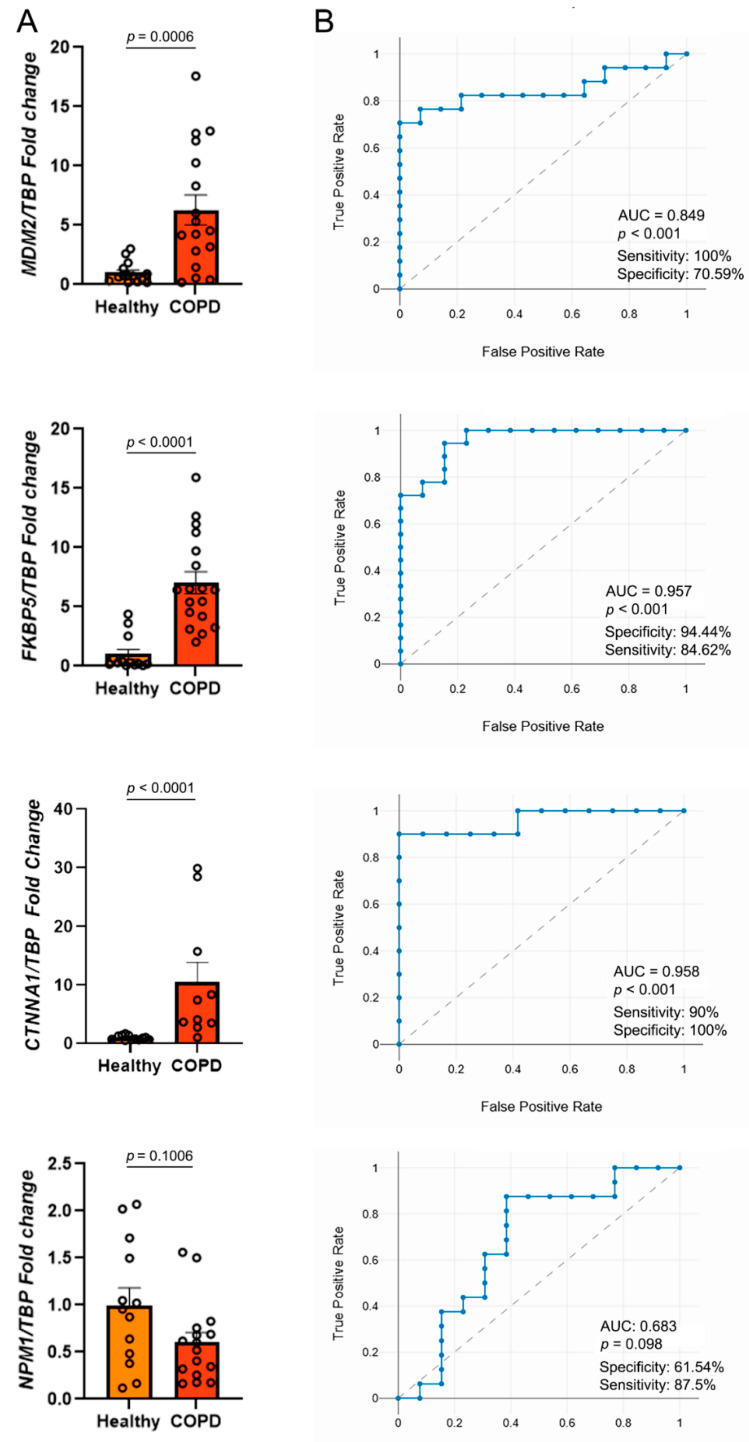
Expression of genes identified by bioinformatics analysis as probable COPD biomarkers in PBMCs of COPD patients. (**A**) RT-qPCR data for healthy volunteers and COPD patients. Expression levels were normalized to the expression levels of *TATA-box binding protein (TBP)* (used as the reference gene). The data are shown as mean ± SEM. The statistical analysis was performed using the Mann–Whitney test. (**B**) ROC (receiver operating characteristic) plot demonstrating the suitability of selected genes as biomarkers; AUC—area under curve.

The expression levels of the six candidate genes were measured via RT-qPCR in PBMCs from our independent cohort of 28 COPD patients and 13 healthy controls. Relative expression was normalized to healthy control levels, which were set to 1. The results of this analysis are summarized in Figure 3.

A significant up-regulation was observed for *MDM2* (6.3-fold, *p* < 0.001), *FKBP5* (7.0-fold, *p* < 0.001), and *CTNNA1* (10.0-fold, *p* < 0.0001) in COPD patients compared to healthy controls (Figure 3A). In contrast, *NPM1* showed a non-significant trend towards down-regulation (approximately 0.6-fold), which is consistent with its identification as the only gene uniformly down-regulated in the bioinformatics analysis (Figure 3A). Analysis of the remaining candidate genes (*ALOX15B* and *GSN*) did not reveal statistically significant differential expression between the groups.

Receiver operating characteristic (ROC) curve analysis was performed to evaluate the diagnostic potential of the significantly altered genes. *FKBP5* and *CTNNA1* exhibited promising diagnostic performance, demonstrating an area under the curve (AUC) of 0.957 and 0.958, with a sensitivity of 84.62% and 90% and a specificity of 94.44% and 100%, respectively (*p* < 0.001) (Figure 3B). *MDM2* also demonstrated diagnostic potential, with an AUC of 0.849, a sensitivity of 70.59%, and a specificity of 100% (*p* < 0.001) (Figure 3B). While *NPM1* showed high sensitivity (87.5%), its low specificity (61.54%) and modest AUC (0.683) indicate the limited diagnostic utility of this gene for COPD.

### 2.6. Network and Text-Mining Analysis of Validated Biomarkers

**Figure 4:**
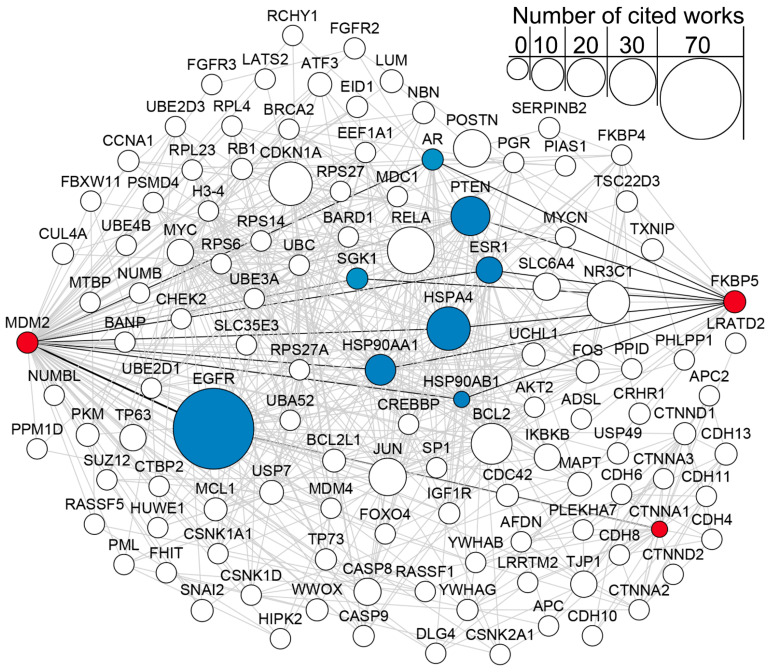
STRING network of the first-order neighbors of potential prognostic genes for COPD (marked in red). Blue nodes are first-order neighbors common for at least two of the genes. Black edges indicate connections between potential prognostic genes through first-order neighbors. The size of the node demonstrates the number of scientific works mentioning the gene and COPD together.

Finally, to understand how the identified genes *MDM2*, *FKBP5*, and *CTNNA1* are interconnected within the COPD-associated regulome, we performed a detailed analysis of the PPI network reconstructed from the DEGs of the GSE146560 dataset. Network analysis revealed that these three genes are interconnected, with *MDM2* and *FKBP5* sharing seven first-order neighbors (*AR*, *PTEN*, *SGK1*, *HSPA4*, *HSP90AA1*, *HSP90AB1*, and *ESR1*) while *CTNNA1* and *MDM2* shared a single first-order neighbor, *EGFR* (Figure 4).

Subsequently, we performed a text-mining analysis to quantify the co-citation of these genes with COPD in the scientific literature. This analysis identified *EGFR* (Epidermal Growth Factor Receptor) as the most frequently referenced gene and *PTEN* and *HSPA4* as the second most cited genes in the context of COPD among all the first-neighbor interactors. The association between the identified genes and known players in COPD pathophysiology further substantiates the potential role of *MDM2*, *FKBP5*, and *CTNNA1* as relevant diagnostic biomarkers.

## 3. Discussion

The need for reliable diagnostic biomarkers for chronic obstructive pulmonary disease (COPD) is driven by the urgent need for earlier detection and a deeper understanding of its underlying molecular mechanisms. During COPD development, clinical signs often do not manifest until a substantial proportion (up to ~ 75%) of lung structure has already been damaged [28], creating a pressing need for molecular tools that can detect the disease in its nascent stage [29].

*Murine double minute 2 (MDM2)* is a key negative regulator of the tumor suppressor p53, involved in the development of cellular stress and senescence, one of the key links in COPD pathogenesis [30]. The p53 pathway is activated in response to the common COPD risk factors, such as cigarette smoke and environmental factors that induce oxidative stress and DNA damage [31]. Under such chronic insults, sustained *MDM2* overexpression could represent an adaptation mechanism to suppress p53-mediated apoptosis and senescence in the airway epithelium. Potentially, by suppressing apoptosis, elevated *MDM2* expression could contribute to the state of low-grade inflammation and impaired lung regeneration ability observed in COPD lungs [32]. Thus, detected up-regulation of *MDM2* may not only be a marker of ongoing cellular stress, but also a potential early indicator of lung environment dysregulation that precedes airway remodeling, suggesting its potential as a diagnostic marker in COPD.

*FK506-binding protein 51 (FKBP5)* is a negative modulator of the glucocorticoid receptor complex, lowering the binding affinity of cortisol and the efficiency of receptor nuclear translocation [33]. The up-regulation of *FKBP5* in COPD patients may suggest a disruption within the stress response axis that *FKBP5* is a part of. Constantly increased expression levels could contribute to a heightened inflammatory state by reducing the efficiency of endogenous cortisol signaling, possibly fostering an environment that sustains the persistent inflammation characteristic of COPD [33]. Moreover, there has been reports indicating that *FKBP5* up-regulation is accociated with worse asthma control in the population of obese children [34], while in a COPD context the single nucleotide polymorphism of this gene (rs4713916) was found to be associated with higher quality of life in COPD patients [35], and functional outcomes [36], indicating the relevance of this gene both in the broader context of respiratory diseases as well as COPD. Thus, measuring *FKBP5* expression levels could identify a state of altered glucocorticoid responsiveness and stress vulnerability, potentially providing an early indication of disease.

*Catenin Alpha 1* (*CTNNA1*) is a central component of adherens junctions, crucial for maintaining epithelial and endothelial barrier integrity as well as cell–cell adhesion [37]. Despite being typically associated with structural functions, changes in its expression in PBMCs have been reported before in the context of vascular [38] and ischemic [39] diseases. However, specific evidence of *CTNNA1* up-regulation in PBMCs of COPD patients has been limited. In the context of COPD, increased *CTNNA1* expression could reflect altered cell plasticity or epithelial-to-mesenchymal transition activation implicated in COPD development [40]. Additionally, there are reports indicating that several immune cell populations, not just PBMCs, are involved in the development of COPD-associated small airway remodeling and fibrosis [41]. Thus, the up-regulation of *CTNNA1* may indicate a role in pathological crosstalk between circulating immune cells and the compromised lung environment, potentially contributing to the chronic inflammatory cycle in COPD.

**Figure 5:**
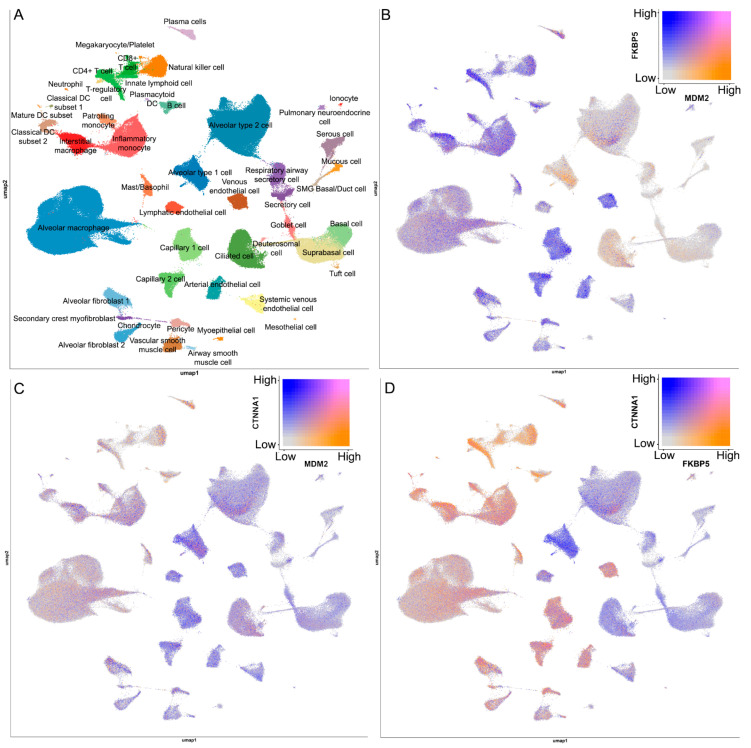
Localization and co-expression of potential COPD prognostic genes in healthy human lungs according to the ShinyCell Human Lung CellRef resource [42]. (**A**) Cell types in healthy human lungs. (**B–D**) Localization and co-expression of *MDM2/FKBP5* (**B**), *MDM2/CTNNA1* (**C**), and *FKBP5/CTNNA1* (**D**).

To elucidate the lung tissue co-expression and cellular localization of the candidate genes (*MDM2*, *FKBP5*, and *CTNNA1*), we analyzed single-cell RNA-sequencing data from the human lung cell atlas provided by the ShinyLung CellRef resource [42] (Figure 5). An overview of the major cell types present in the lung parenchyma is presented in Figure 5A. Analysis of the *MDM2/FKBP5, CTNNA1/MDM2* and *CTNNA1/FKBP5* pairs in these data revealed distinct expression patterns for each gene pair. For the *MDM2*/*FKBP5* pair, *FKBP5* expression was highest in T-cell subsets, interstitial macrophages, and vascular cells (capillary, arterial, and venous endothelial cells). In contrast, *MDM2* showed its most prominent expression in alveolar epithelial type I cells (AEC I) (Figure 5B). In the *CTNNA1*/*MDM2* pair, *CTNNA1* expression was elevated in AEC I and AEC II, while *MDM2* exhibited moderate expression in alveolar macrophages and lymphoid cells (T and B cells) (Figure 5C). Comparison of *CTNNA1* and *FKBP5* showed that *CTNNA1* was most expressed in AEC I and AEC II, whereas *FKBP5* expression was highest in CD4+ T cells, inflammatory monocytes, and macrophage populations (interstitial and alveolar) (Figure 5D). This exploratory analysis indicates that the expression of *MDM2*, *FKBP5*, and *CTNNA1* is not restricted to a single cell type but is detectable across multiple pulmonary cell populations, including structural, immune, and vascular compartments relevant to COPD pathology.

**Figure 6:**
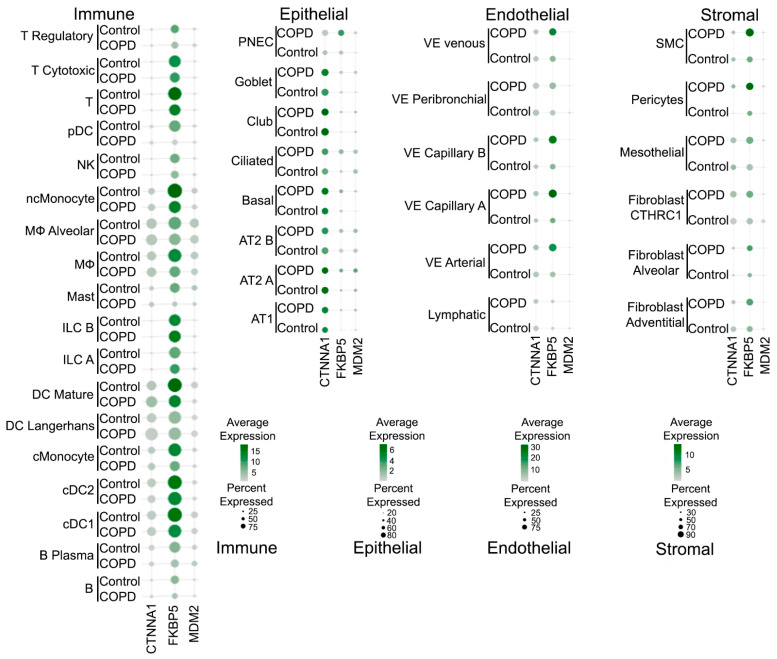
Expression profiles of potential prognostic COPD genes in several subpopulations of lung-affiliated cells in COPD patients versus healthy controls. Data obtained from the COPD cell atlas, published by Sauler et al. [43].

To further confirm the expression dynamics of *MDM2*, *FKBP5*, and *CTNNA1* within the lung microenvironment during disease, we analyzed single-cell RNA-sequencing data from a published COPD cell atlas [43] (Figure 6). This analysis revealed distinct expression changes for each gene in specific cellular compartments in COPD compared to healthy tissue. *FKBP5* expression was significantly elevated in vascular cells, including smooth muscle cells and pericytes, as well as venous, arterial, and capillary endothelial cells. *CTNNA1* showed its most pronounced up-regulation in epithelial populations, particularly in goblet and club cells, while maintaining high expression levels in AECs I and II similar to healthy lungs. In contrast, *MDM2* did not show widespread high expression but demonstrated increased levels in specific immune cell subsets, such as alveolar macrophages, mature dendritic cells, and plasma cells.

These data indicate that the expression patterns of *MDM2*, *FKBP5*, and *CTNNA1* are altered in a cell-type-specific manner within COPD lungs, highlighting their association with structural and immune compartments relevant to disease pathogenesis.

**Figure 7:**
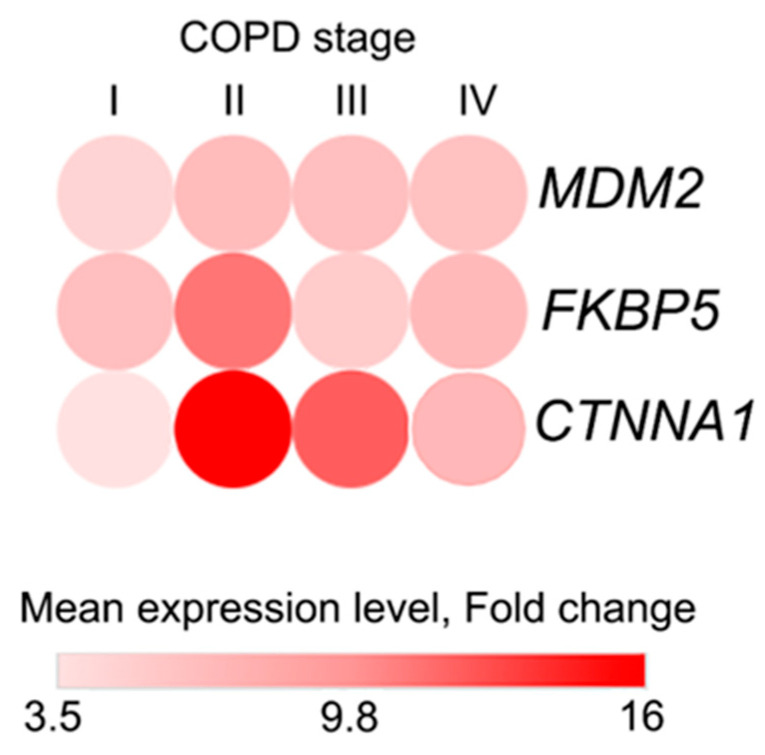
Heatmap demonstrating the changes in mean expression levels of validated genes in COPD patients according to their COPD GOLD stage.

Next, in order to verify the diagnostic value of the identified genes depending on the stage of disease development, we examined the relationship between the expression levels of validated genes and COPD stage according to GOLD criteria. It was found that this relationship was not linear. We observed a steady increase in expression levels of *FKBP5* and *CTNNA1* from Stage I to Stage II, while *MDM2* expression levels remained at the same level. This was followed by a noticeable decline in patients with COPD stages III and IV (Figure 7).

The observed expression pattern suggests the biological processes involving these genes may be most pronounced during early-to-moderate disease. This phase likely represents a period of active cellular stress and inflammatory signaling prior to the onset of severe, irreversible lung function decline. Consequently, these biomarkers could hold particular value for identifying COPD in its earlier stages, when therapeutic interventions may have a greater potential to modify the disease course.

### Limitations of This Study

This article has several limitations. The most obvious limitation is the small number of recruited patients, meaning that the findings of this article should be validated in a bigger cohort of a more heterogeneous patient population. Prospective longitudinal studies in at-risk populations (e.g., heavy smokers with normal spirometry) are needed to validate if these markers can truly predict the future development of COPD. The second major limitation is the lack of detailed clinical characterization of the patients, indicating that future validation in larger, prospectively recruited cohorts with comprehensive clinical data is necessary to confirm the generalizability of our findings. Furthermore, validation in independent cohorts, including those with other respiratory diseases, is essential to confirm diagnostic specificity.

## 4. Materials and Methods

### 4.1. Dataset Selection and Preprocessing

Two independent microarray datasets were retrieved from the National Center for Biotechnology Information Gene Expression Omnibus (NCBI GEO) database (https://www.ncbi.nlm.nih.gov/geo/ (accsessed on 17 March 2025)). The dataset GSE146560 was selected for Weighted Gene Co-Expression Network Analysis (WGCNA) due to its sufficient sample size, 4 samples in four groups, namely, Healthy non-smokers, Healthy smokers, COPD-ex smokers, and COPD smokers, 16 samples in total, and source tissue (PBMCs). The dataset GSE94916, comprising PBMC samples from an independent cohort of healthy controls and COPD patients, was selected for cross-validation. Differentially expressed gene analysis for GSE94916 was performed using the GEO2R online tool with default parameters, and genes with *p* < 0.05 and |log_2_ (fold change)| > 0.585 were selected for downstream analysis.

### 4.2. Weighted Gene Co-Expression Network Analysis (WGCNA)

WGCNA was performed on the GSE146560 dataset using the WGCNA package v. 1.73 [44,45] in RStudio software v. 2025.09.1+401 [46] to identify modules of highly co-expressed genes with potential prognostic value in COPD.

First, we performed a data quality control check. Genes and samples with an excessive number of missing values were filtered out using the goodSamplesGenes function. A sample dendrogram was constructed to identify and remove outlier samples. A soft-thresholding power was selected using the pickSoftThreshol function to construct a scale-free co-expression network. A power of 13 was chosen as the lowest value for which the scale-free topology fit index reached 0.8 and retained sufficient mean connectivity (Figure S1). Subsequently, an adjacency matrix was calculated using the selected soft-thresholding power and transformed into a Topological Overlap Matrix (TOM). The TOM dissimilarity (1-TOM) was used for hierarchical clustering of genes with average linkage. Gene modules were identified from the resulting dendrogram using the cutreeDynamic function with a minimum module size of 30 genes. Module eigengenes (MEs), defined as the first principal component of a given module, were calculated. Modules with a correlation greater than 0.75 were merged, resulting in 11 distinct modules for downstream analysis.

The relationship between modules and the clinical trait of COPD (coded as 0 for healthy controls and 1 for COPD patients) was assessed by calculating the Pearson correlation between module eigengenes and the trait. For genes within significant modules, we calculated gene significance (GS), defined as the absolute value of the correlation between the gene and the COPD trait, and module membership (MM), defined as the correlation between the gene expression profile and the module eigengene.

### 4.3. Functional Enrichment and Protein-Protein Interaction (PPI) Network Analysis

Functional annotation of genes from key modules of interest was performed using the ClueGO v2.5.9 plugin [47] in Cytoscape v3.10.3 [48]. Gene Ontology (GO) biological processes, Kyoto Encyclopedia of Genes and Genomes (KEGG) pathways, Reactome Pathways and WikiPathways were analyzed using a two-sided hypergeometric test with Bonferroni step-down correction. Terms with a corrected p-value < 0.05 were considered significantly enriched. To reconstruct protein–protein interaction (PPI) networks, gene lists were uploaded to the STRING database (https://string-db.org/), and the resulting interaction networks with a confidence score > 0.7 were imported and visualized in Cytoscape.

### 4.4. Patient Cohort and Ethical Approval

A total of 28 COPD patients admitted to the Novosibirsk Second State Clinical City Hospital, as well as 13 age- and gender-matched healthy volunteers, were enrolled in this study. Inclusion, exclusion, and non-inclusion criteria are presented in Table S3. All patients and volunteers who agreed to participate in this study gave written informed consent. During this study, no patients were excluded due to the presence of exclusion criteria or retraction of informed consent. No severe adverse effects due to participation in this study were reported. This study was approved by the Ethics Committee of Novosibirsk State Medical University (protocol No. 121 from 21 November 2019). This study was conducted in compliance with the ethical principles of the Declaration of Helsinki in the current version [49].

### 4.5. PBMC Isolation and RNA Extraction

Peripheral blood samples were collected from all participants in BD Vacutainer tubes (BD Biosciences, Franklin Lakes, NJ, USA) via venipuncture. Peripheral blood mononuclear cells (PBMCs) were isolated within a few hours of collection by density gradient centrifugation using Ficoll-Paque PREMIUM (1.077 g/mL; Pan-Eco, Moscow, Russia) according to the manufacturer’s protocol. Briefly, blood was diluted with saline, layered onto Ficoll, and centrifuged at 400× *g* for 30 min at room temperature. The PBMC layer was carefully aspirated, washed twice with saline, and centrifuged at 300× *g* for 10 min. Cell viability and count were assessed using trypan blue staining. Isolated PBMCs were then lysed in 1 mL TRIzol reagent (Ambion, Carlsbad, CA, USA) for total RNA extraction, which was performed according to the manufacturer’s instructions. RNA concentration and purity were verified spectrophotometrically using a NanoDrop One^C^ Microvolume UV-Vis Spectrophotometer (ThermoFisher Scientific, Waltham, MA, USA).

### 4.6. Quantitative Real-Time PCR (RT-qPCR)

Complementary DNA (cDNA) was synthesized from 2.5 µg of total RNA in a 100 µL reaction mixture using 250 U of M-MuLV-RH reverse transcriptase (Biolabmix, Novosibirsk, Russia) and 100 µM of random hexamer primers. The reaction conditions were 25 °C for 10 min, 42 °C for 60 min, and 70 °C for 10 min. Quantitative PCR was performed in a 25 µL reaction volume containing 5 µL of cDNA, 12.5 µL of 2× HS-qPCR master mix (Biolabmix, Russia), and gene-specific primers and probes. The TATA-box binding protein (TBP) gene was used as an endogenous control. Primer and probe sequences are listed in Table 1. Amplification was carried out on a CFX96 Touch Real-Time PCR Detection System (Bio-Rad Laboratories Inc., Hercules, CA, USA) under the following conditions: initial denaturation at 94 °C for 2 min, followed by 50 cycles of 94 °C for 10 s and 60 °C for 30 s. Gene expression was quantified using the comparative ∆∆Ct method, normalized to TBP. RT-qPCR results were visualized using GraphPad Prism software v.10.4.0 (GraphPad Software, Boston, MA, USA).

### 4.7. ROC Analysis

Receiver operating characteristic (ROC) analysis was performed to assess the diagnostic value of selected genes with respect to COPD diagnosis. ROC curves were constructed using MedCalc software v. 23.3.7 (MedCalc Software, Ostend, Belgium), and the area under the curve (AUC) reflecting model quality was evaluated as follows: 0.9–1.0—excellent, 0.8–0.9—very good, 0.7–0.8—good, 0.6–0.7—moderate, and 0.5–0.6—bad. The model was considered statistically significant at *p*-value < 0.05, area under the curve > 0.5, and sensitivity and specificity > 60%.

### 4.8. Data Mining Analysis

To analyze the co-occurrence of genes of interest and keywords associated with COPD in the scientific texts deposited in the MEDLINE database, we performed a text-mining analysis using the GenCLIP3 web service [50]. Genes of interest were uploaded into GenCLIP3, and a search of co-occurrence between them and the following keywords was performed: COPD, chronic obstructive pulmonary disease, chronic bronchitis, and Emphysema.

### 4.9. Statistical Analysis

Statistical analyses for clinical validation were performed using GraphPad Prism version 10.4.0. Due to the sample size and the potential for non-normal distribution, differences in gene expression between COPD patients and healthy controls were assessed using the non-parametric Mann–Whitney U test. A two-tailed *p*-value of less than 0.05 was considered statistically significant. Data are presented as the mean ± standard error of the mean (SEM). The specific statistical tests used for the bioinformatics components (WGCNA, GEO2R, ClueGO) are described in their respective sections above.

## 5. Conclusions

In conclusion, our findings position *MDM2*, *FKBP5*, and *CTNNA1* as promising diagnostic biomarkers for COPD, reflecting certain aspects of the disease’s pathophysiology: dysregulated cellular stress response and inherent glucocorticoid signaling dysfunction. Moving forward, validating this molecular signature could help in the development of a diagnostic panel that not only identifies COPD but also provides insight into the dominant pathogenic drivers in an individual patient, ultimately facilitating earlier intervention and a more personalized approach to management.

## Figures and Tables

**Table 1:** Sequences of the primers used in this study.

Gene	Type	Sequence
*MDM2*	Forward	5′-AAGGAGAGCAATTAGTGAGACAG-3′
	Probe	5′-((5,6)-FAM)-TTGTGGCGTTTTCTTTGTCGTTCACC-BHQ1-3′
	Reverse	5′-CCCTTATTACACACAGAGCCAG-3′
*ALOX15B*	Forward	5′-TTCTCCAAGGGCTTCCTAAAC-3′
	Probe	5′-((5,6)-FAM)-TGACATACTGCACCAGGGCTTCC-BHQ1-3′
	Reverse	5′-TCCAAGCACAGGAGTCAAAC-3′
*NPM1*	Forward	5′-AAGTATATCTGGAAAGCGGTCTG-3′
	Probe	5′-((5,6)-FAM)-CCCTGGAGGTGGTAGCAAGGTTC-BHQ1-3′
	Reverse	5′-TTTTGGCTGGAGTATCTCGTATAG-3′
*FKBP5*	Forward	5′-CAGTCTCCCTAAAATTCCCTCG-3′
	Probe	5′-((5,6)-FAM)-CCCTCTCCTTTCCGTTTGGTTCTCC-BHQ1-3′
	Reverse	5′-TTGCTCCTTCGTTTGGATTTG-3′
*GSN*	Forward	5′-GCTGGATGACTACCTGAACG-3′
	Probe	5′-((5,6)-FAM)-CCGACTCGAAGCCCTGGACC-BHQ1-3′
	Reverse	5′-TGAATCCTGATGCCACACC-3′
*CTNNA1*	Forward	5′-GAGATGACAGACTTTACCCGAG-3′
	Probe	5′-((5,6)-FAM)-TCCTGGATCCTGCCTCAGCAATTT-BHQ1-3′
	Reverse	5′-GCAATGGTCTGCAATGGTG-3′
*TBP*	Forward	5′-GATAAGAGAGCCACGAACCAC-3′
	Probe	5′-((5,6)-ROX)-CACAGGAGCCAAGAGTGAAGAACAGT-BHQ2-3′
	Reverse	5′-CAAGAACTTAGCTGGAAAACCC-3′

## Data Availability

The original contributions presented in this study are included in the article/Supplementary Materials. Further inquiries can be directed to the corresponding author.

## Supplementary Materials

The following supporting information can be downloaded at https://www.mdpi.com/article/10.3390/ijms27010273/s1.
