# Supplementary material for: A Combined Bioinformatics and Clinical Validation Study Identifies MDM2, FKBP5 and CTNNA1 as Diagnostic Gene Signatures for COPD in Peripheral Blood Mononuclear Cells
Source: Int J Mol Sci. 2025 Dec 26;27(1):273. doi: 10.3390/ijms27010273 (PMC12785598; doi:10.3390/ijms27010273)
Supplement: Supplementary file 1 [file ijms-27-00273-s001.zip › Table S3.pdf]

Table S3. Inclusion, exclusion and non-inclusion criteria for the patients.

Inclusion criteria
• Signed written informed consent to participate in the study<br>• Negative qRT-PCR result for SARS-CoV-2 RNA from nasopharyngeal and oropharyngeal swabs, as well as negative ELISA result for SARS-CoV-2 spike protein specific IgM and IgG in serum at the time of screening<br>• Males and females aged 40 to 65 years<br>• A diagnosis of COPD consistent with the GOLD 2011 criteria (post-bronchodilator ratio of forced expiratory volume in 1 second [FEV1] to forced vital capacity [FVC] less than or equal to 0.7).
Non-inclusion criteria
• Failure to provide informed consent for participation in the study.<br>• Presence of other bronchopulmonary diseases (patients with simple bronchitis or bronchial asthma were eligible).<br>• History of lung resection or lung volume reduction surgery.<br>• Diseases or conditions other than COPD which are associated with eosinophilia.<br>• Inflammatory diseases other than COPD, including autoimmune and infectious diseases.<br>• Malignant neoplasms, regardless of location.<br>• Chronic left-sided ventricular heart failure, stages IIA, IIB, or III.<br>• Stage 5 of chronic kidney disease.<br>• Liver cirrhosis class B or C according to the Child-Pugh classification.<br>• Presence of contraindications to the diagnostic procedures specified in the study protocol.
Exclusion criteria
• Patient's decision to stop their participation in the study<br>• Development of chronic bronchopulmonary diseases other than COPD during the study period (development of simple bronchitis or bronchial asthma was allowed).<br>• Lung resection or lung volume reduction surgery during the study.<br>• Development of diseases or conditions other than COPD associated with eosinophilia during the study.<br>• Development of inflammatory diseases other than COPD, including autoimmune and infectious diseases, during the study.<br>• Emergence of contraindications to the diagnostic procedures specified in the study protocol.
