# Supplementary figures and images for: A Combined Bioinformatics and Clinical Validation Study Identifies MDM2, FKBP5 and CTNNA1 as Diagnostic Gene Signatures for COPD in Peripheral Blood Mononuclear Cells
Source: Int J Mol Sci. 2025 Dec 26;27(1):273. doi: 10.3390/ijms27010273 (PMC12785598; doi:10.3390/ijms27010273)

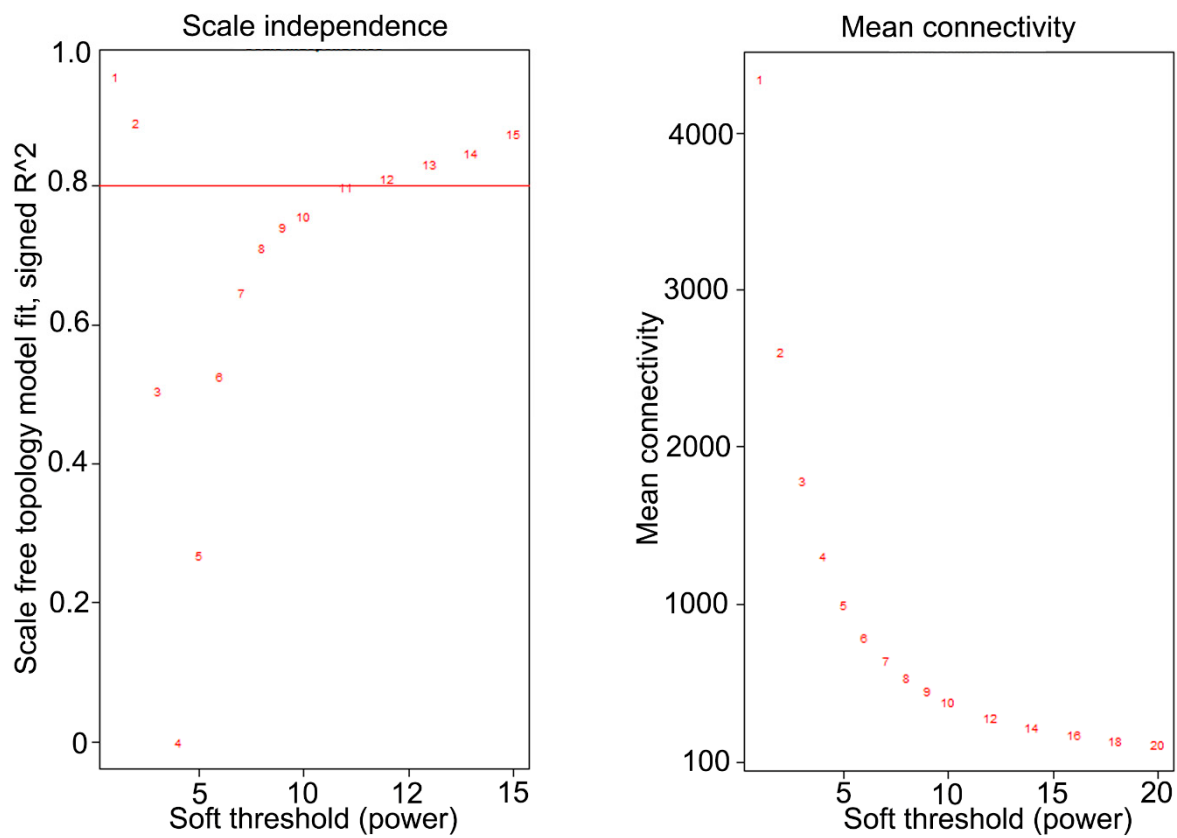

**Figure S1.** Choosing soft-threshold power threshold for the WGCNA.

Supplement: Supplementary file 1 [file ijms-27-00273-s001.zip › Figure S1.pdf]

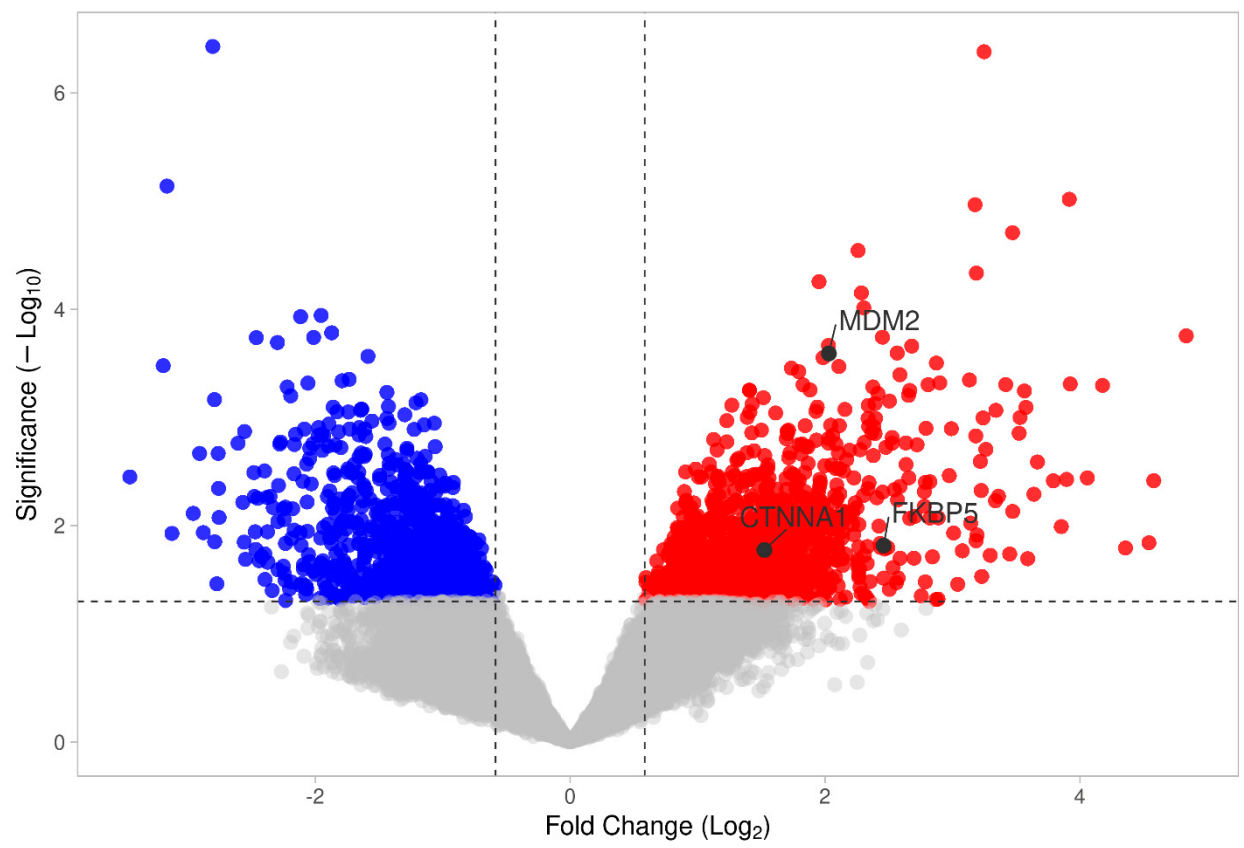

**Figure S2.** Volcano plot of DEGs in GSE94916.

Supplement: Supplementary file 1 [file ijms-27-00273-s001.zip › Figure S2.pdf]
